# Hypothalamic transcriptomic alterations in male and female California mice (*Peromyscus californicus*) developmentally exposed to bisphenol A or ethinyl estradiol

**DOI:** 10.14814/phy2.13133

**Published:** 2017-02-14

**Authors:** Sarah A. Johnson, William G. Spollen, Lindsey K. Manshack, Nathan J. Bivens, Scott A. Givan, Cheryl S. Rosenfeld

**Affiliations:** ^1^ Bond Life Sciences CenterUniversity of MissouriColumbiaMissouri; ^2^ Biomedical SciencesUniversity of MissouriColumbiaMissouri; ^3^ Animal SciencesUniversity of MissouriColumbiaMissouri; ^4^ Informatics Research Core FacilityUniversity of MissouriColumbiaMissouri; ^5^ DNA Core FacilityUniversity of MissouriColumbiaMissouri; ^6^ Molecular Microbiology and ImmunologyUniversity of MissouriColumbiaMissouri; ^7^ Genetics Area ProgramUniversity of MissouriColumbiaMissouri; ^8^ Thompson Center for Autism and Neurobehavioral DisordersUniversity of MissouriColumbiaMissouri

**Keywords:** Brain, DOHaD, endocrine‐disrupting chemicals, estrogens, RNA‐seq

## Abstract

Bisphenol A (BPA) is an endocrine‐disrupting chemical (EDC) prevalent in many household items. Rodent models and human epidemiological studies have linked this chemical to neurobehavior impairments. In California mice, developmental exposure to BPA results in sociosexual disorders at adulthood, including communication and biparental care deficits, behaviors that are primarily regulated by the hypothalamus. Thus, we sought to examine the transcriptomic profile in this brain region of juvenile male and female California mice offspring exposed from periconception through lactation to BPA or ethinyl estradiol (EE, estrogen present in birth control pills and considered a positive estrogen control for BPA studies). Two weeks prior to breeding, P_0_ females were fed a control diet, or this diet supplemented with 50 mg BPA/kg feed weight or 0.1 ppb EE, and continued on the diets through lactation. At weaning, brains from male and female offspring were collected, hypothalamic RNA isolated, and RNA‐seq analysis performed. Results indicate that BPA and EE groups clustered separately from controls with BPA and EE exposure leading to unique set of signature gene profiles. *Kcnd3* was downregulated in the hypothalamus of BPA‐ and EE‐exposed females, whereas *Tbl2*,* Topors*,* Kif3a*, and *Phactr2* were upregulated in these groups. Comparison of transcripts differentially expressed in BPA and EE groups revealed significant enrichment of gene ontology terms associated with microtubule‐based processes. Current results show that perinatal exposure to BPA or EE can result in several transcriptomic alterations, including those associated with microtubule functions, in the hypothalamus of California mice. It remains to be determined whether these genes mediate BPA‐induced behavioral disruptions.

## Introduction

Exposure to endocrine‐disrupting chemicals (EDCs), including bisphenol A (BPA), may increase the risk of neurobehavioral disorders typified by social impairments, such as autism spectrum disorders (Grandjean and Landrigan 2006; Landrigan 2010; Dietert et al. 2011; Miodovnik et al. 2011; Braun 2012; de Cock et al. 2012; Braun et al. 2014; Kalkbrenner et al. 2014; Kaur et al. 2014; Stein et al. 2015). Most EDCs are manufactured chemicals (Diamanti‐Kandarakis et al. 2009) with BPA being one of the most ubiquitous (He et al. 2009; Biedermann et al. 2010; Galloway et al. 2010). In 2013, production of this chemical was estimated to be approximately 15 billion pounds (GrandViewResearch, 2014). Its stability and pervasiveness (Environment Canada, 2008) has ensured continual exposure (Vandenberg et al. 2009). This chemical is detectable in the urine of 93% of the U.S. population (Calafat et al. 2008), as well as in fetal plasma, placenta (vom Saal et al. 2007), and breast milk (Vandenberg et al. 2007). In 2012, the FDA banned the production of baby bottles and sippy cups containing BPA https://www.federalregister.gov/documents/2012/07/17/2012-17366/indirect-food-additives-polymers. (accessed 23 January 2017). However, this restriction fails to address transfer of BPA across the placenta and through the milk (Ikezuki et al. 2002; Kawamoto et al. 2007; Balakrishnan et al. 2010; Nishikawa et al. 2010; Vandenberg et al. 2010). Moreover, fetuses and neonates lack many enzymes needed to metabolize BPA and may experience greater levels of active BPA than the mother (Ikezuki et al. 2002; Kawamoto et al. 2007; Nishikawa et al. 2010).

The fetus is especially vulnerable to endocrine disruption. Sex steroid hormones guide brain sexual differentiation throughout gestation and into the neonatal period (Arnold and Breedlove 1985; Adkins‐Regan 2005; McCarthy 2008). Environmental chemicals that mimic or interfere with sex hormone action may disturb this development (Palanza et al. 1999; Jasarevic et al. 2012; Kundakovic et al., 2013b). Broad ranges of behaviors are influenced by BPA exposure. Affected behaviors include reproductive, emotional, cognitive, and social behaviors and spatial reasoning (Berenbaum and Hines 1992; Mueller et al. 2008; Puts et al. 2008; Geary 2010). Many of the sociosexual deficits associated with BPA exposure in children and animals models are reviewed in Rosenfeld (2015). In our own studies, we tested two *Peromyscus* species, one that is polygynous and female uniparental (deer mice, *P. maniculatus bairdii*) and the other that is monogamous and biparental (California mice, *Peromyscus californicus*). In a mate choice experiment, females selectively reject deer mice males developmentally exposed to BPA (Jasarevic et al. 2011). Adult male California mice perinatally exposed to BPA show reduced territorial marking, a form of communication needed to protect the home range and mate from intruders, whereas exposed females demonstrate decreased exploratory and voluntary physical activity behaviors (Williams et al. 2013; Johnson et al. 2015c). Both male and female California mice developmentally exposed to BPA or ethinyl estradiol (EE, estrogen present in birth control pills) exhibit compromised parental care (Johnson et al. 2015b). These adult behavioral disruptions likely trace their origins to disturbances in normal brain programming during the perinatal period.

There is strong conservation in brain development and function across taxa, including in rodents and humans (Rice and Barone 2000; Howdeshell 2002). The hypothalamus is one of the primary brain areas governing many of these sociosexual behaviors. Thus, BPA might induce global transcriptomic changes in this brain region that is essential for guiding sociosexual behaviors. By using in situ hybridization, cDNA expression array, Northern blot, and qPCR approaches, prior rodent and zebrafish (*Danio rerio*) animal model, and in vitro cell culture studies report that BPA can alter individual candidate genes in hypothalamic regions or isolated neurons (Funabashi et al. 2001; Ceccarelli et al. 2007; Fukushima et al. 2007; Monje et al. 2007; Cao et al. 2012, 2013, 2014; Kundakovic et al., 2013a; Warita et al. 2013, 2014; Chen et al. 2014; Cano‐Nicolau et al. 2016). Collectively, these studies suggest BPA disrupts the hypothalamic expression of aromatase B (*Cyp19a1b)*, nerve growth factor (*Ngf*), glucocorticoid receptor (*Gr*), estrogen receptor α (*Esr1* and transcript variants), estrogen receptor β (*Esr2*), DNA methyltransferases (*Dnmt1*,* 3a, 3b*), methyl‐CpG‐binding protein 2 (*Mecp2*), kisspeptin (*Kiss1*), transforming growth factor‐β3 (*Tgfb3*), and progesterone receptor (*Pr*). A recent study suggests that in utero exposure of rats to BPA can induce select gene expression differences on postnatal day (PND) 1 (Arambula et al. 2016). However, to our knowledge, no previous studies have examined the comprehensive transcriptomic profile changes in the hypothalamus of a rodent model exposed to BPA or EE throughout the pre‐ and postnatal period, with the latter period approximating the third trimester of neural development in the hypothalamus, hippocampus, amygdala, and other brain regions in humans (Rice and Barone 2000; Howdeshell 2002). Such transcriptomic alterations may provide useful biomarkers of early exposure to these EDC. With this notion in mind, we used RNA‐seq to determine the global transcriptomic alterations in the hypothalamus of weanling (30 days of age) male and female California mice exposed to BPA throughout gestation and lactation. This time period was chosen as it represents the end of the exposure period to these chemicals and reflects the hypothalamic gene expression patterns prior to the observed adult‐onset behavioral deficits. The hypotheses at the outset were that (1) developmental exposure through the maternal diet to BPA or EE would induce global gene expression changes in the hypothalamus of juvenile California mice and (2) BPA and EE transcriptomic alterations would be dependent on offspring sex.

## Materials and Methods

### Animals and treatments

Founder out‐bred adult (60–90 days of age) California mice females and males were purchased from the *Peromyscus* Genetic Stock Center (PGSC) at the University of South Carolina (Columbia, SC), and placed in quarantine for a minimum of 8 weeks to ensure that they did not carry any common rodent pathogens. At the PGSC, *P. californicus* captive stocks have been bred to maintain their out‐bred status. We currently have our own breeding colony at the University of Missouri. As needed, additional California mice are purchased to maintain their out‐bred status. All experiments were approved by the University of Missouri Animal Care and Use Committee (Protocol #8693). Experiments were performed in accordance with the recommendations in the Guide for the Care and Use of Laboratory Animals of the National Institutes of Health. Two weeks prior to breeding, virgin females, 8–12 weeks of age were randomly assigned to receive one of three diets: (1) a low phytoestrogen AIN 93G diet supplemented with 7% by weight corn oil to minimize potential phytoestrogenic contamination that would otherwise be present with inclusion of soybean oil in the diet, (2) the same diet supplemented with 50 mg BPA/kg feed weight, which we have documented to lead to internal serum concentrations close to those measured in pregnant women unknowingly exposed to this chemical (Jasarevic et al. 2011; Sieli et al. 2011), or (3) AIN93G diet supplemented with 0.1 parts per billion of EE, as the U.S. Food and Drug Administration (FDA) required estrogen‐positive control for BPA studies (vom Saal et al. 2005; Johnson et al. 2015a). The FDA has requested EE be included in BPA studies that may guide policy decisions based on the premise that BPA acts primarily as a weak estrogen (Vandenberg et al. 2009). The diets were started two weeks prior to breeding to span the periconceptional period. Females were maintained on these diets throughout gestation and lactation, as described previously (Jasarevic et al. 2011, 2013; Williams et al. 2013). To avoid any potential litter effects, only one male and one female offspring per litter were randomly chosen and examined in the current studies. At weaning, five replicates of each sex and group were euthanized, and brains were flash frozen on dry ice, and stored at −80°C until the hypothalamus was dissected, as described previously for mice (Kundakovic et al., 2013a). While no brain dissection guide for *Peromyscus* is available, the landmarks described in the Rat and Mouse Brain Dissection Guide Atlases (Paxinos and Franklin 2008; Paxinos 2013) can be used for California mice.

### RNA isolation from hypothalamic samples

Hypothalamic RNA was isolated using Qiagen AllPrep DNA/RNA/miRNA Universal kit (Qiagen, Valencia, CA). Isolated DNA and miRNA will be used in future studies. The quantity and quality of the RNA was determined using a Nanodrop ND1000 spectrophotometer (Nanodrop Products, Wilmington, DE). The results were further confirmed by analyzing the RNA on the Fragment Analyzer (Advanced Analytical Technologies, Ankeny, IA). Only RNA with a RQN score above 8.0 was used for RNA‐seq analyses. Five different animals representing five different litters per each sex and each group were initially tested such that a total of 50 mice were initially screened. Similar numbers of replicates in other species have been successfully used to delineate transcriptomic changes following exposure to other environmental chemicals (Richter et al. 2014; Wood et al. 2014; Wirbisky et al. 2015; Arambula et al. 2016).

### Illumina TruSeq RNA library preparation and sequencing

High‐throughput sequencing was performed at the University of Missouri DNA Core Facility. Libraries were constructed following the manufacturer's protocol with reagents supplied in Illumina's TruSeq Stranded mRNA Library Preparation kit. Briefly, the poly‐A containing mRNA is purified from total RNA (2 μg), RNA is fragmented, double‐stranded cDNA is generated from fragmented RNA, and the index containing adapters are ligated to fragment ends. PCR amplification was performed as follows: 98°C^(0:30)^ + [98°C^(0:10)^ + 60°C^(0:30)^ + 72°C^(0:30)^] × 15 cycles + 72°C^(5:00)^. The amplified cDNA construct were purified with Axyprep Mag PCR Clean‐up beads. Purified libraries were evaluated using the Fragment Analyzer (Advanced Analytical Technologies, Ankeny, IA), quantified with the Qubit 2.0 Flurometer (Invitrogen, Carlsbad, CA) using the quant‐iT HS dsDNA reagent kit, and diluted according to Illumina's standard sequencing protocol for sequencing on the HiSeq 2500 with a single end, 50 base read length. To maximize the number of reads per sample, only three samples were included in each lane, and the groups of three samples were randomized across treatments to avoid any confounding effects due to sequencing lane.

### Gene expression analyses

Previously described methods (Givan et al. 2012) were modified to create a custom transcriptome and determine differential transcriptome expression. Specifically, 50‐mer RNA‐Seq reads from the Illumina HiSeq were first cleaned using scripts from the Fastx Toolkit ( https://github.com/agordon/fastx_toolkit) to trim the 3' ends of low‐quality (phred score < 20) bases and dropping reads when less than 50 bases were remaining. Reads were then filtered to exclude those that did not have a minimum of 95% of their bases with a quality score of 20 or more. Adapter sequence was removed with CutAdapt (Martin 2011) version 1.8.3. To create a final set of quality controlled RNA‐seq reads, foreign or undesirable reads were removed by sequence matching to the Phi‐X genome ( https://www.ncbi.nlm.nih.gov/genome/?term=NC_001422.1), the relevant ribosomal RNA genes as downloaded from the National Center for Biotechnology Information (NCBI; https://www.ncbi.nlm.nih.gov) or repeat elements in RepBase version 20.02 (Jurka et al. 2005) using Bowtie version 1.1.1 (Langmead et al. 2009). Trinity version trinityrnaseq_r20140717 (Haas et al. 2013) was used to assemble the quality controlled reads into transcripts using default parameters. The final set of assembled transcripts is available for download ( https://genomics.ircf.missouri.edu/cgi-psd/ppsd.cgi?db=681). It is common to use paired‐end DNA sequence reads to de novo assemble transcriptomes. However, a priority was to balance the cost of the experiments with generating the data of most value. For this work, generating multiple replicates per sample of gene expression data was of higher value than generating a comprehensive transcript assembly. And it has been demonstrated that using single‐end (SE) reads for transcriptome assembly and quantification has relatively minor effects on gene‐level DE estimates, as described previously (Gonzalez and Joly 2013; Chhangawala et al. 2015). Thus, we chose to use SE data for this work. Functional annotation of the assembled transcripts was done by identifying proteins with significant sequence similarity within the NCBI NR sequence database using BLAST (Altschul et al. 1997).

To investigate the relationships between sample replicates, we first identified the list of genes that were differentially expressed (DE) between any two conditions of interest (DE genes). We collected the gene expression levels of all DE genes in each sample replicate. Upon visualizing the relationships between sample replicates based on the DE genes via a hierarchical cluster plot, samples from the EE group, EE.M1 and EE.M2, appeared to be significant outliers. To test the possibility that these samples were legitimate outliers and could be removed from the analysis, we first generated a distance matrix of the expression values of the DE genes between all sample replicates. The mean distance values were calculated for all sample replicates. Samples EE.M1 and EE.M2 had the highest mean distance from all other sample replicates. Upon removing these two values from the list of mean distances, the remaining distribution of values satisfied the Anderson–Darling test for normality, *P* = 0.06, as implemented in the nortest package in R ( https://cran.r-project.org/web/packages/nortest/index.html). Subsequently, the distance values for EE.M1 and EE.M2 were determined to be outliers using the Grubbs test for two outliers, *P* = 2.2e^−16^, as implemented in the outliers package in R ( https://cran.r-project.org/web/packages/outliers/index.html). Therefore, samples EE. M1 and EE.M2 were removed from all subsequent analyses discussed below. Subsequent to the removal of EE.M1 and EE.M2, no additional outliers were detected using the Grubbs test. Additionally, results from one of the EE female replicate samples were discarded due to 3X lower yield during the library generation. Lower yield may be due to a variety of reasons, but the potential for bias is high, and thus, this sample was not considered in subsequent analyses.

Differential expression of the transcripts in the remaining replicates was determined using RSEM version 1.2.15 (Li and Dewey 2011) and EdgeR version 3.8.2 (Robinson et al. 2010), as implemented in TrinityRNAseq. A transcript was considered differentially expressed between two conditions if the FDR value associated with the expression ratio was <0.05. Visualization of the gene expression results via the 3D plot of the principal component analysis was done using ggplot2 version 2.1.0 (Springer‐Verlag, 2009) and rgl version 0.96.0 (Adler 2016). Visualization of the differential expression via the heatmap was done using the ComplexHeatmap (Gu et al. 2016) package. Functional assessments of sets of differentially expressed genes such as pathway analyses and enrichment analyses were done using the TargetMine website (Chen et al. 2016). Before doing functional analyses, de novo‐assembled transcripts were mapped to human proteins based on extended amino acid similarity.

## Results

### General characterizations

The goal was to generate ~50 million reads per sample by loading three samples to each lane. The average number of raw reads was 59,965,113 and mapped reads was 43,462,565. Table 1 summarizes the alignment of RNA‐seq reads for each individual sample to the *P. californicus* genome sequence. For all three groups (BPA, EE, and controls) and both sexes, similar results were obtained in all of the categories. This number of reads and gene annotations is considered more than sufficient for a eukaryotic genome (Givan et al. 2012).

**Table 1:** Summary of California mice hypothalamic RNA‐seq data generated and aligned to the reference genome sequence

Sample type	Sample	Raw reads	QC	Mapped reads	Mapping efficiency
Control ♀	1	69,130,197	55,971,358	51,865,339	92.7
	2	59,953,540	46,892,283	43,166,939	92.1
	3	63,537,487	49,756,404	46,269,276	93
	4	56,255,792	43,270,695	40,186,046	92.9
	5	70,576,520	54,790,689	50,805,216	92.7
Control ♂	1	46,376,849	34,835,622	33,238,813	95.4
	2	65,673,113	51,651,522	47,823,627	92.6
	3	52,258,679	42,073,456	38,938,021	92.6
	4	59,220,633	47,041,870	43,660,840	92.8
	5	39,935,256	31,793,528	29,287,635	92.1
BPA ♀	1	45,592,976	36,777,665	33,894,109	92.2
	2	62,081,241	48,332,836	44,626,692	92.3
	3	48,640,791	37,908,444	34,912,852	92.1
	4	64,901,425	49,432,382	45,486,404	92
	5	71,890,086	56,217,073	51,563,272	91.7
BPA ♂	1	66,308,474	49,724,724	45,792,562	92.1
	2	56,296,984	44,408,114	40,927,183	92.2
	3	62,267,149	49,653,807	45,765,399	92.2
	4	68,054,969	53,980,003	50,117,318	92.8
	5	80,868,525	63,580,564	58,320,110	91.7
EE ♀	1	55,370,002	43,910,820	40,531,812	92.3
	2	65,056,368	50,734,415	46,898,849	92.4
	3	56,097,553	42,583,645	39,390,432	92.5
	4	54,602,416	42,752,931	39,532,155	92.5
EE ♂	1	60,772,693	48,331,755	44,729,572	92.6
	2	51,359,489	40,459,368	37,291,216	92.2
	3	65,978,847	52,139,890	48,467,572	93

### Gene expression, association, and pathway differences

**Figure 1:**
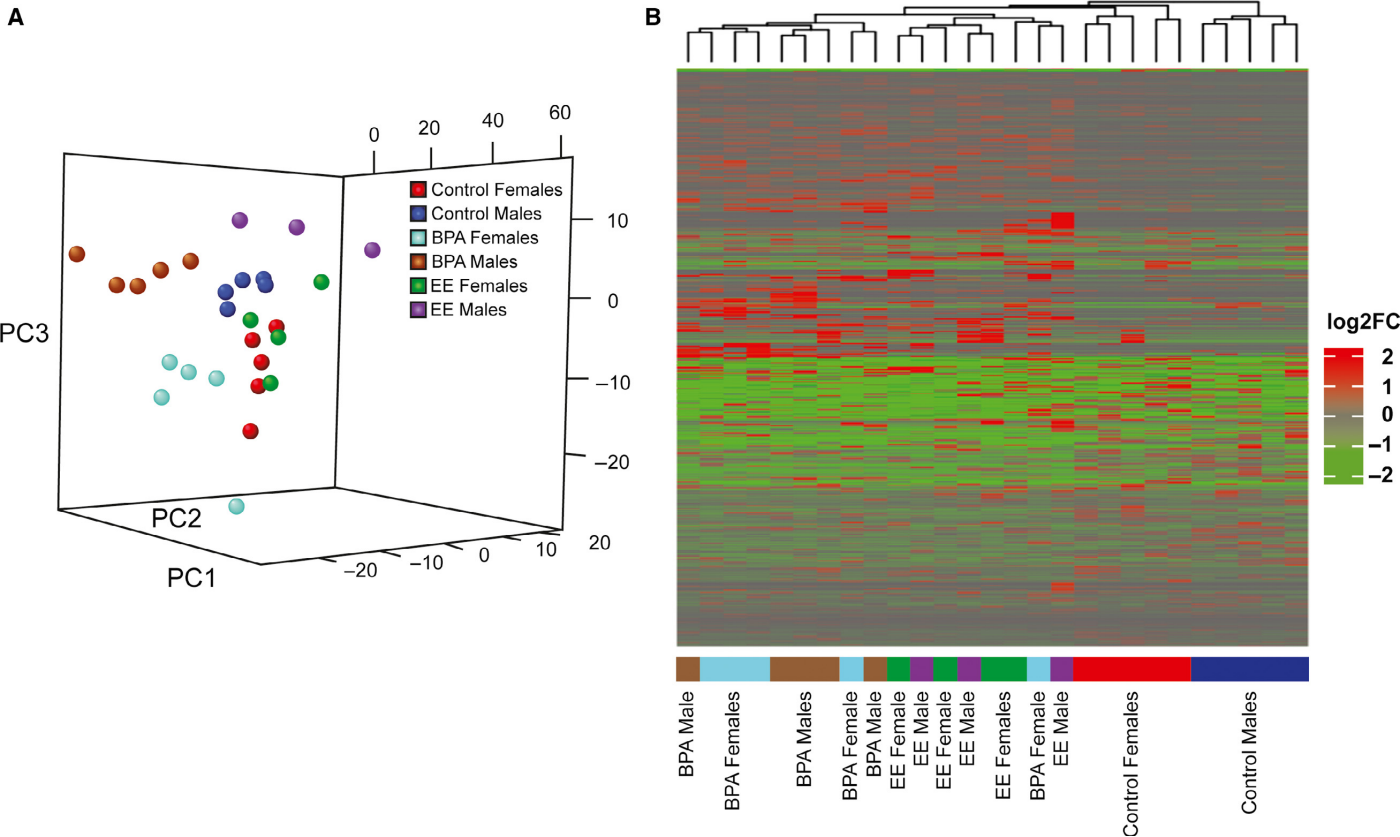
PCA plot and hierarchical heatmap. (A) PCA plot of the FPKM values of differentially expressed genes shows a nonrandom distribution of points (PERMANOVA = 2e^−04^ from 10,000 permutations), but control and EE females clustered together. (B) Hierarchical clustering and heatmap of expression ratios for the differentially expressed genes reveals primarily four groups: BPA‐treated, EE‐treated, control male, and control female groups.

The PCA plot based on FPKM values shows a nonrandom distribution of points associated with samples (PC1 24.6%, PC2 10.2%, PC3 9.7%, PERMANOVA = 2e^−04^ from 10,000 permutations; Fig. 1A). Groupings of points are relatively obvious for BPA‐treated males, BPA‐treated females, EE‐treated males, and control males, whereas control females and EE‐treated females are intermingled. Even though the EE female samples cluster with the control female samples, it does not necessarily mean that the effects of EE on the female hypothalamus transcriptome are minor as further detailed below. A heatmap based on the hierarchical clustering of expression ratios for the differentially expressed genes revealed four main clusters containing primarily samples of BPA treatment, EE treatment, control females, and control males (Fig. 1B). There was no clear separation based on sex in either the BPA‐ or EE‐treated samples.

**Figure 2:**
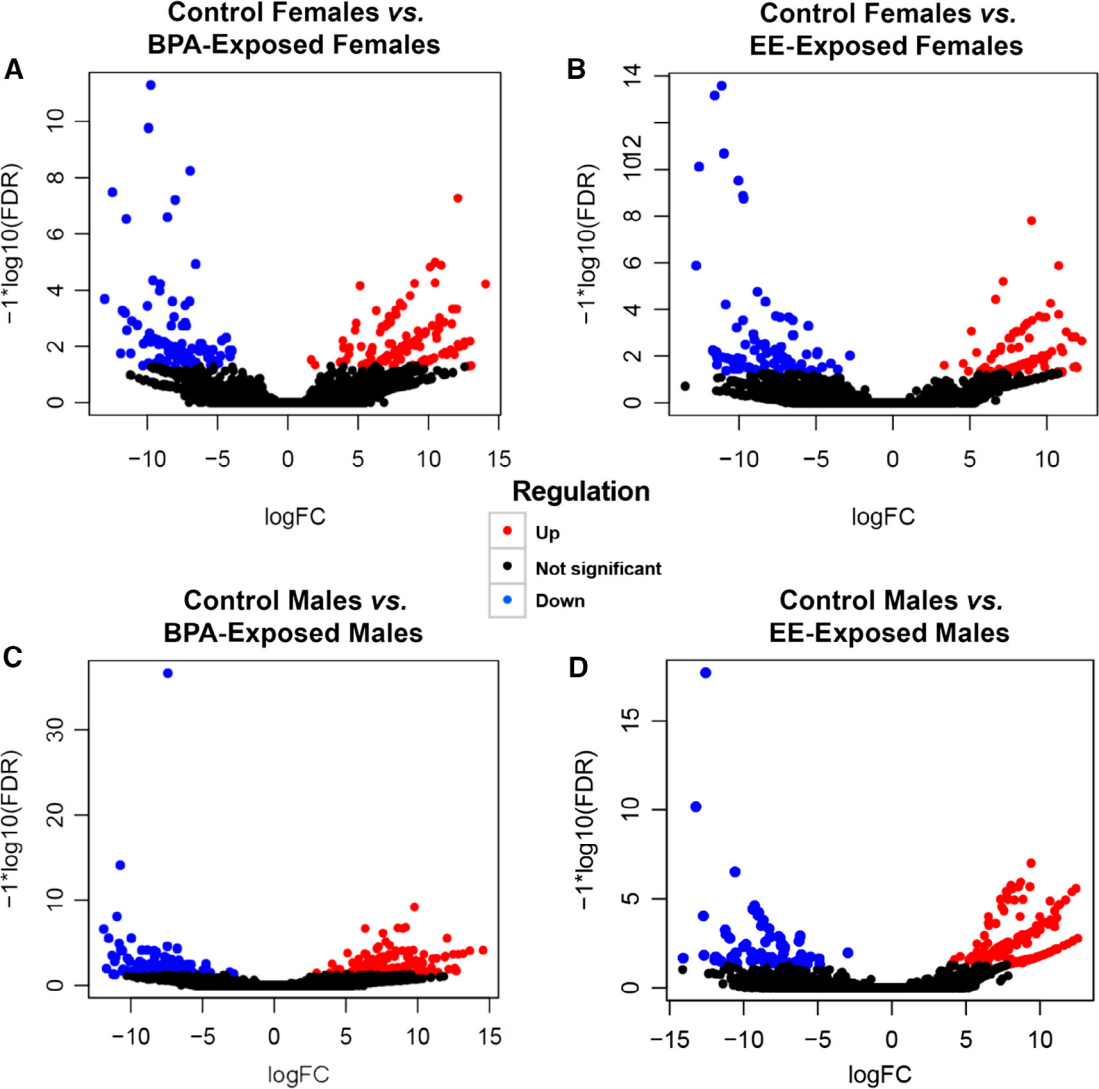
Volcano plots. (A) Control females versus BPA‐exposed females. (B) Control females versus EE‐exposed females. (C) Control males versus BPA‐exposed males. (D) Control males versus EE‐exposed males. Significantly downregulated genes in the treatment group versus controls are depicted in blue, black genes indicate that they are not significantly different, and genes delineated in red are increased in expression relative to controls.

**Figure 3:**
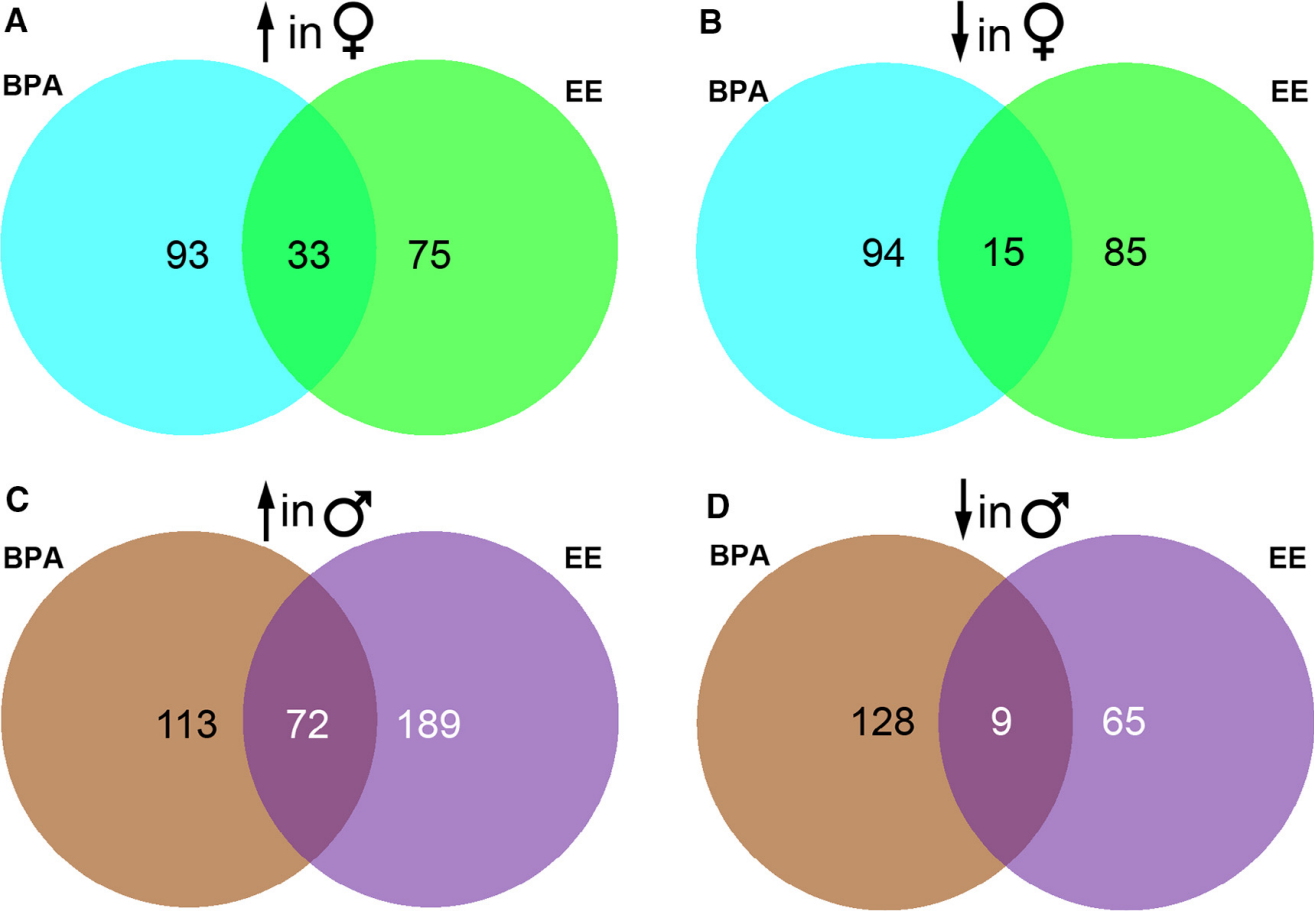
Venn diagrams. (A) Transcripts upregulated in BPA‐exposed females versus control females compared to those upregulated in EE‐exposed females versus control females. (B) Transcripts downregulated in BPA‐exposed females versus control females compared to those downregulated in EE‐exposed females versus control females. (C) Transcripts upregulated in BPA‐exposed males versus control males compared to those upregulated in EE‐exposed males versus control males. (D) Transcripts downregulated in BPA‐exposed males versus control males compared to those upregulated in EE‐exposed males versus control males.

Volcano plot comparisons revealed that our approach led to sufficient coverage to detect gene expression differences in BPA‐ or EE‐exposed males and females relative to their respective control groups (Fig. 2). Venn diagram comparison for females revealed select genes that were either increased or decreased in both BPA‐ and EE‐exposed individuals (Fig. 3A and B, Supporting Information File 1). However, each group possessed unique signature profile of genes that include transcripts that were increased and decreased in both groups. Likewise, BPA‐ and EE‐exposed males had select genes that were up‐ and downregulated in both groups (Fig. 3C and D, Supporting Information File 1), but more genes were either increased or decreased in one of the two treatment groups.

As described earlier, EdgeR was used to identify the genes that differed in the following comparisons: control females versus control males, control females versus BPA females, control females versus EE females, control males versus BPA males, control males versus EE males, BPA females versus BPA males, and EE females versus EE males (Supporting Information File 2). Sex differences in hypothalamic gene expression between the three treatment groups are further addressed below. Tables 2 and 3 show the top 20 genes that are down‐ and upregulated, respectively, in BPA females versus control females. Tables 4 and 5 include the top 20 genes that are down‐ and upregulated, respectively, in EE females versus control females. Tables 6 and 7 show the top 20 genes that are down‐ and upregulated, respectively, in BPA males versus control males. Tables 8 and 9 include the top 20 genes that are down‐ and upregulated, respectively, in EE males versus control males. These collective tables indicate that each Treatment × Sex combination led to a unique signature set of genes. In other words, different genes were differentially regulated by BPA and EE and these depend on offspring sex. The exception is *Kcnd3*, which was downregulated in the hypothalamus of BPA‐ and EE‐exposed females relative to control females (bold in Tables 2 and 4). *Tbl2*,* Topors*,* Kif13a*, and *Phactr2* were in the top 20 genes upregulated in BPA‐ and EE‐exposed females relative to control females (bold in Tables 3 and 5). No genes were shared in the top 20 genes differentially regulated in both BPA‐ and EE‐exposed males versus control males. Supporting Information File 3 provides gene differences between the three comparisons (BPA vs. control, EE vs. control, and BPA vs. EE) that were in common for both sexes.

**Table 2:** Top 20 annotated genes downregulated in BPA‐exposed females compared to control females

Entrez ID	Gene symbol	Gene name	FDR	Log2 fold change
9524	TECR	Very long‐chain enoyl‐CoA reductase isoform 2	0.0002	−13.043
114928	GPRASP2	Gprasp1 protein	3.236E‐08	−12.493
170506	DHX36	ATP‐dependent RNA helicase DHX36	0.0178	−11.872
4594	MUT	Methylmalonyl‐CoA mutase, mitochondrial	0.0005	−11.768
23683	PRKD3	Serine/threonine‐protein kinase D3 isoform 1	0.0006	−11.574
90627	STARD13	stAR‐related lipid transfer protein 13 isoform 2	2.865E‐07	−11.475
25828	TXN2	Thioredoxin, mitochondrial precursor	0.0026	−11.454
1741	DLG3	Disks large homolog 3 isoform 2	0.0171	−11.179
**3752**	**KCND3**	**Potassium voltage‐gated channel subfamily D member 3 isoform 1 precursor**	**0.0013**	**−11.092**
65109	UPF3B	Regulator of nonsense transcripts 3B isoform X2 (*Peromyscus maniculatus bairdii*)	0.0017	−10.696
23633	KPNA6	Importin subunit *α*‐7	0.0480	−10.303
2342	FNTB	Protein farnesyltransferase subunit beta isoform X2 (*Macaca fascicularis*)	0.0079	−10.281
8910	SGCE	Epsilon‐sarcoglycan isoform X4 (*Peromyscus maniculatus bairdii*)	0.0004	−9.9968
54906	FAM208B	protein FAM208B isoform X1 (*Peromyscus maniculatus bairdii*)	0.0055	−9.9953
9663	LPIN2	*Mus musculus* strain C57BL6/J chromosome 6 clone RP23‐258N2, complete sequence	1.66E‐10	−9.9061
501	ALDH7A1	*α*‐Aminoadipic semialdehyde dehydrogenase isoform X1 (*Peromyscus maniculatus bairdii*)	0.0034	−9.8387
287	ANK2	Ankyrin‐2 (*Propithecus coquereli*)	0.0059	−9.7829
55759	WDR12	Ribosome biogenesis protein WDR12 (*Peromyscus maniculatus bairdii*)	0.0067	−9.7639
4124	MAN1A1	Mannosyl‐oligosaccharide 1,2‐*α*‐mannosidase IA isoform X1 (*Peromyscus maniculatus bairdii*)	0.0474	−9.7301
81614	NIPA2	Magnesium transporter NIPA2 (*Peromyscus maniculatus bairdii*)	0.0430	−9.6497

**Table 3:** Top 20 annotated genes upregulated in BPA‐exposed females compared to control females

Entrez ID	Gene symbol	Gene name	FDR	Log2 fold change
**26608**	**TBL2**	***Microtus ochrogaster*** **guanine nucleotide binding protein (G protein), gamma 2 (Gng2), mRNA**	**5.84E‐05**	**14.060**
23008	KLHDC10	Kelch domain‐containing protein 10	0.0473	13.042
170506	DHX36	ATP‐dependent RNA helicase DHX36 isoform X2	0.0067	12.949
157378	TMEM65	Transmembrane protein 65 (*Microtus ochrogaster*)	0.0071	12.571
10938	EHD1	EH‐domain containing 1, partial	0.0089	12.542
3920	LAMP2	Lysosome‐associated membrane glycoprotein 2 isoform 2 precursor	0.0092	12.409
4301	MLLT4	Afadin isoform X15	0.0102	12.165
9026	HIP1R	Huntingtin‐interacting protein 1‐related protein isoform X1 (*Mesocricetus auratus*)	0.0086	12.098
221937	FOXK1	Forkhead box protein K1	5.23E‐08	12.075
**10210**	**TOPORS**	***Mustela putorius*** **furo UDP‐Gal:betaGlcNAc beta 1,4‐ galactosyltransferase, polypeptide 5 (B4GALT5), partial mRNA**	**0.0148**	**11.784**
**63971**	**KIF13A**	**Kinesin‐like protein KIF13A isoform X4 (** ***Peromyscus maniculatus bairdii*** **)**	**0.0004**	**11.690**
23274	CLEC16A	Protein CLEC16A isoform X4	0.0015	11.641
129049	SGSM1	Small G protein signaling modulator 1 isoform X1 (*Mesocricetus auratus*)	0.0133	11.569
**9749**	**PHACTR2**	**Phosphatase and actin regulator 2 isoform X1**	**0.0160**	**11.394**
55731	FAM222B	Protein FAM222B isoform X1 (*Peromyscus maniculatus bairdii*)	0.0161	11.172
253959	RALGAPA1	Ral GTPase‐activating protein subunit *α*‐1 isoform X4	0.018	10.986
8314	BAP1	Ubiquitin carboxyl‐terminal hydrolase BAP1	1.29E‐05	10.90
375	ARF1	*Peromyscus maniculatus bairdii* PHD finger protein 20‐like 1 (Phf20l1), transcript variant X3, mRNA	0.0009	10.741
10401	PIAS3	E3 SUMO‐protein ligase PIAS3 (*Microtus ochrogaster*)	0.001	10.780
26035	GLCE	D‐glucuronyl C5‐epimerase (*Peromyscus maniculatus bairdii*)	0.0009	10.557
113402	SFT2D1	*Peromyscus maniculatus bairdii* SFT2 domain containing 1 (Sft2d1), mRNA	0.0051	10.592

**Table 4:** Top 20 annotated genes downregulated in EE‐exposed females compared to control females

Entrez ID	Gene symbol	Gene name	FDR	Log2 fold change
22864	R3HDM2	R3H domain‐containing protein 2 isoform X9	1.33E‐06	−12.787
23017	FAIM2	Protein lifeguard 2 isoform 1	7.67E‐11	−12.591
9671	WSCD2	WSC domain‐containing protein 2	0.0055	−11.717
3149	HMGB3	High‐mobility group protein B3 isoform X2	6.54E‐14	−11.604
5905	RANGAP1	Ran GTPase‐activating protein 1 isoform X3	0.0067	−11.488
54982	CLN6	*Peromyscus maniculatus bairdii* CDP‐diacylglycerol‐inositol 3‐phosphatidyltransferase (Cdipt), transcript variant X2, mRNA	0.0075	−11.451
10390	CEPT1	Choline/ethanolaminephosphotransferase 1 isoform 1	0.0225	−11.439
59338	PLEKHA1	Pleckstrin homology domain‐containing family A member 1 isoform X9	0.0109	−11.437
9915	ARNT2	Aryl hydrocarbon receptor nuclear translocator 2 isoform X1	2.57E‐14	−11.130
**3752**	**KCND3**	**Potassium voltage‐gated channel subfamily D member 3 isoform 1 precursor**	**0.0109**	**−11.056**
23378	RRP8	Ribosomal RNA‐processing protein 8 isoform 2	2.05E‐11	−10.974
7690	ZNF131	Zinc finger protein 131, isoform CRA_a	5.85E‐05	−10.866
113829	SLC35A4	UDP‐sugar transporter protein SLC35A4	0.0412	−10.820
140609	NEK7	*Microtus ochrogaster* NIMA‐related kinase 7 (Nek7), mRNA	0.0116	−10.817
10206	TRIM13	E3 ubiquitin‐protein ligase TRIM13	0.0126	−10.684
745	MYRF	Myelin regulatory factor isoform X6	0.0158	−10.410
84915	FAM222A	Protein FAM222A	0.0154	−10.371
4728	NDUFS8	NADH dehydrogenase (ubiquinone) iron‐sulfur protein 8, mitochondrial	0.0198	−10.334
2186	BPTF	Nucleosome‐remodeling factor subunit BPTF isoform X6	0.0348	−10.330
1973	EIF4A1	Eukaryotic initiation factor 4A‐I (*Mesocricetus auratus*)	0.0348	−10.289

**Table 5:** Top 20 annotated genes upregulated in EE‐exposed females compared to control females

Entrez ID	Gene symbol	Gene name	FDR	Log2 fold change
**26608**	**TBL2**	***Microtus ochrogaster*** **guanine nucleotide binding protein (G protein), gamma 2 (Gng2), mRNA**	**0.0022**	**12.258**
78986	DUSP26	Dual specificity protein phosphatase 26	0.0303	11.953
64400	AKTIP	AKT‐interacting protein isoform 1	0.0271	11.904
5909	RAP1GAP	Rap1 GTPase‐activating protein 1 isoform 2	0.0272	11.887
54467	ANKIB1	*Peromyscus maniculatus bairdii* RAD23 homolog B (*Saccharomyces cerevisiae*) (Rad23b), mRNA	0.0290	11.738
**63971**	**KIF13A**	**Kinesin family member 13A isoform X4**	**0.0015**	**11.621**
8266	UBL4A	*Peromyscus maniculatus bairdii* ubiquitin‐like 4A (Ubl4a), mRNA	0.0067	11.259
113178	SCAMP4	Secretory carrier membrane protein 4, isoform CRA_c	0.0468	10.990
**10210**	**TOPORS**	***Mustela putorius*** **furo UDP‐Gal:betaGlcNAc beta 1,4‐ galactosyltransferase, polypeptide 5 (B4GALT5), partial mRNA**	**0.0472**	**10.973**
10150	MBNL2	*Peromyscus maniculatus bairdii* muscleblind‐like splicing regulator 2 (Mbnl2), transcript variant X2, mRNA	0.0044	10.917
**9749**	**PHACTR2**	**Phosphatase and actin regulator 2 isoform D**	**0.0490**	**10.900**
4311	MME	CD10 neutral endopeptidase 24.11	1.34E‐06	10.778
26035	GLCE	d‐glucuronyl C5‐epimerase	0.0002	10.774
NA	CMPK1	*Cricetulus griseus* cytidine monophosphate (UMP‐CMP) kinase 1, cytosolic (Cmpk1), mRNA	0.044	10.735
100506658	OCLN	Occludin, isoform CRA_b	0.006	10.300
10659	CELF2	CUGBP Elav‐like family member 2 isoform 1	5.40E‐05	10.240
112970	KTI12	Protein KTI12 homolog	0.0089	10.064
23114	NFASC	Neurofascin isoform X12	0.0002	9.9501
2058	EPRS	Bifunctional glutamate/proline–tRNA ligase isoform X1	0.0286	9.8765
346157	ZNF391	Zinc finger protein 282‐like (*Peromyscus maniculatus bairdii*)	0.0174	9.7798

**Table 6:** Top 20 annotated genes downregulated in BPA‐exposed males compared to control males

Entrez ID	Gene symbol	Gene name	FDR	Log2 fold change
125950	RAVER1	*Peromyscus maniculatus bairdii* ribonucleoprotein, PTB‐binding 1 (Raver1), transcript variant X3, mRNA	0.0453	−11.3089
58508	KMT2C	Histone‐lysine N‐methyltransferase 2C isoform X2 (*Mus musculus*)	0.0358	−11.1299
1149	CIDEA	Cell death activator CIDE‐A (*Peromyscus maniculatus bairdii*)	0.0343	−10.9595
146547	PRSS36	*Peromyscus maniculatus bairdii* LysM, putative peptidoglycan‐binding, domain containing 4 (Lysmd4), mRNA	0.0269	−9.9781
11346	SYNPO	Synaptopodin isoform X3 (*Peromyscus maniculatus bairdii*)	0.0271	−9.6247
1307	COL16A1	Collagen *α*‐1(XVI) chain isoform X4 (*Peromyscus maniculatus bairdii*)	7.14E‐7	−9.5970
84893	FBXO18	F‐box DNA helicase 1 isoform X1 (*Peromyscus maniculatus bairdii*)	0.0270	−9.5249
1663	DDX11	Probable ATP‐dependent RNA helicase DDX11 (*Mesocricetus auratus*)	0.0079	−9.4999
6445	SGCG	Gamma‐sarcoglycan (*Peromyscus maniculatus bairdii*)	0.0096	−9.4984
7384	UQCRC1	Cytochrome b‐c1 complex subunit 1, mitochondrial (*Peromyscus maniculatus bairdii*)	0.0352	−9.4551
284098	PIGW	Phosphatidylinositol‐glycan biosynthesis class W protein (*Peromyscus maniculatus bairdii*)	0.0163	−9.4045
131873	COL6A6	Collagen *α*‐4(VI) chain‐like (*Peromyscus maniculatus bairdii*)	0.0407	−9.3181
57698	SHTN1	Shootin‐1 isoform X1 (*Peromyscus maniculatus bairdii*)	0.0005	−9.2885
8732	RGNTT	mRNA‐capping enzyme isoform X4 (*Peromyscus maniculatus bairdii*)	0.0149	−9.2616
2043	EPHA4	Low‐quality protein: ephrin type‐A receptor 10 (*Lipotes vexillifer*)	0.0458	−9.2506
284403	WDR62	WD repeat‐containing protein 62 isoform X4 (*Peromyscus maniculatus*)	0.0250	−9.2319
284111	SLC13A5	Solute carrier family 13 member 5 isoform X1 (*Peromyscus maniculatus*)	0.0252	−9.2208
138881	OR1L8	*Mesocricetus auratus* wntless homolog (Drosophila) (Wls), transcript variant X1, mRNA	0.0085	−9.1619
10114	HIPK3	Homeodomain‐interacting protein kinase 3 isoform X4 (*Peromyscus maniculatus bairdii*)	0.0043	−9.0835
11201	POLI	DNA polymerase iota isoform X3 (*Peromyscus maniculatus bairdii*)	0.0035	−9.0237

**Table 7:** Top 20 annotated genes upregulated in BPA‐exposed males compared to control males

Entrez ID	Gene symbol	Gene name	FDR	Log2 fold change
84239	ATP13A4	Probable cation‐transporting ATPase 13A4 isoform X1 (*Peromyscus maniculatus bairdii*)	1.76E‐07	Double check
26576	SRPK3	SRSF protein kinase 3 isoform X2 (*Peromyscus maniculatus bairdii*)	0.0170	14.5735
583	BBS2	Bardet‐Biedl syndrome 2 protein (*Peromyscus maniculatus bairdii*)	0.0176	13.6189
93099	DMKN	*Peromyscus maniculatus bairdii* dermokine (Dmkn), mRNA	0.0192	13.1855
26053	AUTS2	Autism susceptibility gene 2 protein isoform X5 (*Peromyscus maniculatus bairdii*)	0.0218	12.8210
11335	CBX3	*Peromyscus maniculatus bairdii* AHA1, activator of heat‐shock 90kDa protein ATPase homolog 2 (yeast) (Ahsa2), mRNA	0.0402	12.7135
64837	KLC2	Kinesin light chain 2 (*Peromyscus maniculatus bairdii*)	0.0407	12.5940
222235	FBXL13	F‐box/LRR‐repeat protein 13 isoform X2 (*Cricetulus griseus*)	0.0033	12.5725
10524	KAT5	Histone acetyltransferase KAT5 isoform X1 (*Orcinus orca*)	0.0016	12.4747
9919	SEC16A	Protein transport protein Sec16A isoform X2 (*Peromyscus maniculatus bairdii*)	0.0338	12.1622
79041	TMEM38A	Trimeric intracellular cation channel type A (*Mesocricetus auratus*)	0.0437	12.0578
79041	RTEL1	Regulator of telomere elongation helicase 1 isoform X5 (*Peromyscus maniculatus bairdii*)	0.0156	11.9364
51750	KDM2A	Lysine‐specific demethylase 2A isoform X1 (*Peromyscus maniculatus bairdii*)	0.0112	11.4489
22992	ELMOD3	ELMO domain‐containing protein 3 (*Peromyscus maniculatus bairdii*)	0.0087	11.3341
84173	OTUD7B	OTU domain‐containing protein 7B (*Peromyscus maniculatus bairdii*)	0.0004	11.2632
56957	DIXDC1	Dixin isoform X7 (*Ceratotherium simum simum*)	0.0022	11.0358
85458	PCYOX1	Prenylcysteine oxidase 1 (*Peromyscus maniculatus bairdii*)	0.0450	10.9514
51449	MAPK15	Mitogen‐activated protein kinase 15 isoform X3 (*Peromyscus maniculatus bairdii*)	0.0486	10.9511
225689	SLC6A13	Sodium‐ and chloride‐dependent GABA transporter 2 (*Peromyscus maniculatus bairdii*)	0.0437	10.8735
27148	KDM1B	Lysine‐specific histone demethylase 1B (*Peromyscus maniculatus bairdii*)	0.0133	10.7690

**Table 8:** Top 20 annotated genes downregulated in EE‐exposed males compared to control males

Entrez ID	Gene symbol	Gene name	FDR	Log2 fold change
2805	GOT1	Aspartate aminotransferase	0.0207	−14.0902
23017	FAIM2	Protein lifeguard 2 isoform X2 (*Tupaia chinensis*)	6.19E‐11	−13.2226
6585	SLIT1	Slit homolog 1 protein (*Peromyscus maniculatus bairdii*)	8.74E‐05	−12.7204
51747	LUC7L3	Luc7‐like protein 3 isoform X1 (*Microtus ochrogaster*)	0.0141	−12.6836
152926	PPM1K	Protein phosphatase 1K, mitochondrial (*Peromyscus maniculatus bairdii*)	1.92E‐18	−12.5670
26278	SACS	Sacsin isoform X1 (*Peromyscus maniculatus bairdii*)	0.0248	−11.9637
11237	RNF24	Ring finger protein 24 (*Cricetulus griseus*)	0.0177	−11.9202
3992	FADS1	Fatty acid desaturase 1 isoform X2 (*Peromyscus maniculatus bairdii*)	0.0188	−11.8140
10207	PATJ	InaD‐like protein isoform X4 (*Peromyscus maniculatus bairdii*)	0.0005	−11.2626
6650	CAPN15	Calpain‐15 isoform X1 (*Peromyscus maniculatus bairdii*)	0.0009	−11.2105
22866	CNKSR2	Connector enhancer of kinase suppressor of ras 2 isoform X4 (*Peromyscus maniculatus bairdii*)	0.0017	−10.9313
6196	RPS6KA2	Ribosomal protein S6 kinase *α*‐2 isoform X2 (*Peromyscus maniculatus bairdii*)	0.0171	−10.808
51430	SUCO	Low‐quality protein: SUN domain‐containing ossification factor, partial (*Peromyscus maniculatus bairdii*)	0.0107	−10.5324
10507	SEMA4D	Semaphorin‐4D isoform X4 (*Peromyscus maniculatus bairdii*)	0.0110	−10.5169
23378	RRP8	Ribosomal RNA‐processing protein 8 (*Peromyscus maniculatus bairdii*)	0.0389	−10.4778
124944	C17ORF49	Chromatin complexes subunit BAP18 isoform X3 (*Cricetulus griseus*)	0.0045	−10.0029
5165	PDK3	Pyruvate dehydrogenase kinase, isozyme 3 isoform X2 (*Peromyscus maniculatus bairdii*)	0.0034	−9.8266
84286	TMEM175	Endosomal/lysomomal potassium channel TMEM175 (*Peromyscus maniculatus bairdii*)	0.0270	−9.4840
255967	PAN3	PAB‐dependent poly(A)‐specific ribonuclease subunit PAN3 isoform X8 (*Mus musculus*)	0.0094	−9.4430
126119	JOSD2	Josephin‐2 isoform X1 (*Cricetulus griseus*)	3.58E‐05	−9.3777

**Table 9:** Top 20 annotated genes upregulated in EE‐exposed males compared to control males

Entrez ID	Gene symbol	Gene name	FDR	Log2 fold change
30000	TNPO2	Transportin‐2 (*Mesocricetus auratus*)	0.0016	12.5441
6196	RPS6KA21	Ribosomal protein S6 kinase *α*‐2 isoform X2 (*Peromyscus maniculatus bairdii*)	2.59E‐10	12.4266
2036	EPB41L1	Band 4.1‐like protein 1 isoform X12 (*Peromyscus maniculatus bairdii*)	0.0022	12.2511
84918	LRP11	Low‐density lipoprotein receptor‐related protein 11 (*Peromyscus maniculatus bairdii*)	0.0024	12.1854
386675	KRTAP10‐7	*Cricetulus griseus* protein kinase C, *α* (Prkca), transcript variant X1, mRNA	0.0032	11.8746
84893	FBXO18	F‐box DNA helicase 1 isoform X1 (*Peromyscus maniculatus bairdii*)	1.08E‐05	11.7283
9026	HIP1R	Huntingtin‐interacting protein 1‐related protein isoform X1 (*Mesocricetus auratus*)	0.0041	11.5967
79411	GLB1L	*β*‐Galactosidase‐1‐like protein isoform X1 (*Peromyscus maniculatus bairdii*)	2.05E‐5	11.3220
4043	LRPAP1	*α*‐2‐Macroglobulin receptor‐associated protein (*Peromyscus maniculatus bairdii*)	2.42E‐5	11.2051
56927	GPR108	Protein GPR108 (*Peromyscus maniculatus bairdii*)	0.0061	11.1658
22800	RRAS2	Ras‐related protein R‐Ras2 (*Elephantulus edwardii*)	0.0001	11.1491
113178	SCAMP4	Secretory carrier membrane protein 4, isoform CRA_c	0.0068	11.0335
11346	SYNPO	Synaptopodin isoform X3 (*Peromyscus maniculatus bairdii*)	4.62E‐5	10.9740
51290	ERGIC2	Endoplasmic reticulum‐Golgi intermediate compartment protein 2 isoform X3 (*Microtus ochrogaster*)	0.0004	10.9722
22826	DNAJC8	DnaJ homolog subfamily C member 8 isoform X2 (*Peromyscus maniculatus bairdii*)	0.0002	10.9581
396	ARHGDIA	Rho GDP‐dissociation inhibitor 1 isoform X2 (*Cricetulus griseus*)	0.0090	10.7457
8848	TSC22D1	*Peromyscus maniculatus bairdii* TSC22 domain family, member 1 (Tsc22d1), transcript variant X2, mRNA	0.0001	10.7014
11011	TLK2	Serine/threonine‐protein kinase tousled‐like 2 isoform X1 (*Peromyscus maniculatus bairdii*)	0.0094	10.6909
2887	GRB10	growth factor receptor‐bound protein 10 isoform X2 (*Peromyscus maniculatus bairdii*)	1.25E‐5	10.6643
N/A	N/A	*Peromyscus maniculatus bairdii* ArfGAP with SH3 domain, ankyrin repeat and PH	0.0099	10.6337

Analysis of the gene expression differences to examine for gene ontology (GO) and pathways that were significantly different only revealed one term GO: 0007017: microtubule‐based processes, where there were 42 genes and FDR <0.05. Genes involved in this process were significantly enriched in the EE males compared to control males, and BPA males compared to control males. No other GO terms or pathways were significantly enriched in any of the other comparisons.

### Sex differences in hypothalamic gene expression

Tables S1 and S2 list the top 20 genes that are down‐ and upregulated, respectively, in control males versus control females. Tables S3 and S4 include the top 20 genes that are down‐ and upregulated, respectively, in BPA males versus BPA females. Tables S5 and S6 include the top 20 genes that are down‐ and upregulated, respectively, in EE males versus EE females. Similar to above, sex differences in down‐ and upregulated genes varied based on the treatment. In the case of the top 20 genes that were downregulated in males, the only ones that overlapped include *Celf4* in control and BPA groups (shaded in Tables S1 and S3), and *Kcnip4* in control and EE groups (bold in Tables S1 and S5). However, comparison of the top 20 genes that are downregulated in males compared to females in BPA and EE revealed no overlapping transcripts (Tables S3 and S5). For the top 20 gene that are upregulated in males versus females, *Tbkbp1* was included in the control and BPA groups (shaded in Tables S2 and S4). No genes were shared among the top 20 genes that are upregulated in males versus females for control and EE and BPA and EE groups (Tables S2, S4, and S6).

## Discussion

The goals of the current study were to determine if developmental exposure through the maternal diet to BPA or EE induces global gene expression changes in the hypothalamus of juvenile California mice. Second, we sought to determine whether the BPA and EE transcriptomic alterations were dependent on offspring sex. Since BPA is considered a weak estrogen (Vandenberg et al. 2009), the notion at the outset was that within sex similar gene expression pattern changes would be seen in BPA‐ and EE‐exposed individuals. The final aim was to examine for sexually dimorphic gene expression patterns within the control, BPA, and EE groups to determine if early exposure to one of the EDCs altered naturally occurring sex differences in hypothalamic gene expression in juvenile animals.

In relation to the first goal, RNA‐seq analysis identified several genes that were differentially expressed in the hypothalamus of BPA‐ and EE‐exposed females and males relative to their respective controls. Predictably, the specific gene expression patterns induced by each treatment varied based on offspring sex. A recent study examined gene expression patterns in the hippocampus and hypothalamus of PND1 rats exposed in utero to varying doses of BPA and EE, and reported only minimal gene expression changes (Arambula et al. 2016). As detailed above, this study design, however, failed to consider the postnatal exposure, which may be even more critical in rodent hypothalamic and other brain region development. It is this time period that approximates the third trimester in humans (Rice and Barone 2000; Howdeshell 2002). Additionally, in the previous study, the average number of reads per sample, presumably raw (although not explicitly specified) was only 29.2 million for the hypothalamus, which is about half of the total number of reads that were generated in the current studies. This difference is likely because the earlier study included more samples per lane, although no indication was provided on how many samples per lane were sequenced. In the current study, only three samples were sequenced per lane to ensure sufficient coverage per sample. Of the genes listed in the article, there does not appear to be any overlap with the current studies. Although the authors indicate a full set of differentially expressed hypothalamic genes included in Table S2a and S2b, these could not be accessed from the journal website nor could any of the other supplemental material with the message on this site reading: “We are sorry, this page is not available.” Thus, no additional comparisons to this earlier study can be made until the files become publicly available.

Although select genes were up‐ and downregulated in both BPA and EE females and males, in general, for both sexes unique genes were altered in BPA and EE groups. The findings thus provide further evidence that BPA can induce effects by binding to other steroidogenic and nonsteroidogenic receptors besides ESRs. Another interpretation of these data is that as a weak estrogen, BPA may not fully recapitulate the same gene expression changes as EE.

However, select genes were shared among highly differentially expressed genes in BPA‐ and EE‐exposed females and males. These transcripts will be further considered. *Kcnd3* was among the most highly downregulated genes in the hypothalamus of both BPA‐ and EE‐exposed females. This gene encodes for the voltage‐gated potassium 4.3 (Kv4.3) channel. In humans, mutation of KCND3 is associated with cerebellar ataxia, intellectual disability, epilepsy, attention deficit disorders, and other clinical signs (Smets et al. 2015). Similar to the current findings, estrogen decreases transcriptional expression of Kv4.3 in the myometrium (Song et al. 2001).

Tranducin (beta)‐like 2 (*Tbl2*) was among the most highly upregulated genes for BPA‐ and EE‐exposed females. This gene encodes a protein that localizes to the endoplasmic reticulum (ER). Under ER stress conditions, it interacts with PKR‐like ER‐resident kinase (PERK), modulates protein expression of activating transcription factor 4 (ATF4), and associates with 60S ribosomal subunit with the net effect being alterations of specific proteins (Tsukumo et al. 2015). Topoisomerase I‐binding protein (*Topors*) is also one of the genes that showed the greatest expression increase in BPA‐ and EE‐exposed females. During oxidative stress, TOPRS dissociates from the H2AX protein and exerts an important role in chromatin reorganization during DNA repair and apoptosis (Seong et al. 2012). Little is known about the role of neural *Kif13a*, which is also upregulated in these two treatment groups. Deletion of this kinesin family motor protein in mice results in anxiety‐like behaviors, likely due to reduced neuronal transport of 5HT(1A) receptor (Zhou et al. 2013). *Phactr2* is also upregulated in these two groups. Phosphatase and actin regulators (PHACTRs) are abundant in the brain where they are reported to mediate activity of protein phosphatase 1 and actin‐binding protein. Additionally, *Phactr2* expression is elevated after traumatic brain injury (Kim et al. 2012). Taken together, the genes that are abundant in BPA‐ and EE‐exposed females appear to be ones that in many cases are upregulated under varying stressful conditions and may help to partially alleviate further cellular damage and ensuing behavioral disturbances.

In males, no genes were common among the 20 most highly differentiated genes expressed in both BPA‐ and EE‐exposed males. However, looking outside of these 20 genes, 9 and 72 transcripts were down‐ and upregulated, respectively, in both of these groups (Fig. 2). These shared transcripts are involved in a variety of functions, as shown in Supporting Information File 2.

Comparison of all the gene expression changes in females and males for BPA and EE groups only revealed one GO term to be enriched, microtubule‐based processes. With their pleiotrophic roles, microtubules are essential for proper neuronal function. Microtubules must respond quickly to environmental changes to provide structural support for the Golgi apparatus, axon guidance, dendrites, neurite outgrowth, interkinetic nuclear migration, and separation of chromatids during mitosis to list a few of the functions ascribed to these structures in neurons (Prokop 2013; Breuss and Keays 2014; Liu and Dwyer 2014; Sainath and Gallo 2015). The microtubule cytoskeleton works in conjunction with microfilaments (actin) and intermediate filaments to facilitate intracellular transport. Previous studies suggest that BPA exposure can disrupt microtubule‐associated proteins in hypothalamic neurons (Yokosuka et al. 2008; Iwakura et al. 2010) and other cell types (Nakagomi et al. 2001; Lehmann and Metzler 2004; Can et al. 2005; George et al. 2008). Ethinyl estradiol can also disrupt microtubular function (Epe et al. 1989; Sato et al. 1992).

In relation to the final aim, several genes were identified in all groups to be expressed in a sexually dimorphic pattern. However, there was minimal overlap of genes between the three groups, indicating that each treatment leads to a unique signature pattern within each sex. Alternatively, early exposure to BPA and EE altered the normal pattern of sex‐specific neurobehavioral programming. Other EDCs lead to sex‐dependent changes in the hypothalamus of largemouth bass (*Micropterus salmoides*) (Martyniuk et al. 2010, 2013) and rats (Walker et al. 2014).

While it would have been optimal to confirm the gene expression differences with qPCR, all of the RNA from the micropunch samples was used for the RNA‐seq analysis. Such studies will be performed in follow‐up studies, along with examining for protein expression differences. We also wish to examine other brain regions and non‐neural tissues to determine if there are common transcripts altered by developmental exposure to BPA and EE. Furthermore, it would be of interest to examine for phenotypic changes that are consistent with BPA‐induced microtubule dysfunction.

In conclusion, the current results show that perinatal exposure to BPA or EE mediates sex‐dependent changes in the hypothalamus of juvenile California mice. Furthermore, BPA and EE exposure results in unique signature of hypothalamic transcripts in males and females. The one common target of BPA and EE exposure may be microtubule‐based processes, which could lead to a wide range of pathophysiological effects in neuronal cells. The studies also demonstrate that even in juvenile animals there are normal sexually dimorphic differences in gene expression in the hypothalamus, but these may be disrupted by early exposure to either EDC tested. It remains to be determined whether the gene expression changes, including microtubule‐associated genes, are responsible for behavioral deficits observed in California mice and possibly by translation to humans.

## Conflict of Interest

The authors declare no competing financial interests.

## Supporting information




**Table S1**. Top 20 annotated genes downregulated in control males compared to control females. Shaded row is included in the BPA group (Table S3), whereas bold row is included in the EE group (Table S5).

**Table S2**. Top 20 annotated genes upregulated in control males compared to control females. Shaded row is included in the BPA group (Table 4).

**Table S3**. Top 20 annotated genes downregulated in BPA males compared to BPA females. Shaded row is included in the Control group (Table S1).

**Table S4**. Top 20 annotated genes upregulated in BPA males compared to BPA females. Shaded row is listed in controls (Table S2).

**Table S5**. Top 20 annotated genes downregulated in EE males compared to EE females. Bold row is included in the control group (Table S1).

**Table S6**. Top 20 annotated genes upregulated in EE males compared to EE females.

**Data S1:** *Genes Increased or Decreased in Both BPA‐ and EE‐Exposed Groups*.

**Data S2:** *Differentially Expressed Genes Based on Treatment and Sex*.

**Data S3:** *Differentially Expressed Genes Based on Treatment*.
