# Supplementary material for: Hypothalamic transcriptomic alterations in male and female California mice (Peromyscus californicus) developmentally exposed to bisphenol A or ethinyl estradiol
Source: Physiol Rep. 2017 Feb 14;5(3):e13133. doi: 10.14814/phy2.13133 (PMC5309579; doi:10.14814/phy2.13133)
Supplement: Supplementary file 1 — Table S1. Top 20 annotated genes downregulated in control males compared to control females. Shaded row is included in the BPA group (Table S3), whereas bold row is included in the EE group (Table S5). [file PHY2-5-e13133-s001.docx]

| **Supplementary Table 1**. Top 20 annotated genes down regulated in control males compared to control females. Shaded row is also included in the BPA group (Supplementary Table 3); whereas bolded row is also included in the EE group (Supplementary Table 5). | | | | |
| --- | --- | --- | --- | --- |
| **Entrez ID** | **Gene Symbol** | **Gene Name** | **FDR** | **Log2 Fold Change** |
| 8848 | TSC22D1 | *Peromyscus maniculatus bairdii* TSC22 domain family, member 1 (Tsc22d1), transcript variant X2, mRNA | 8.09E-07 | -12.8867 |
| 4736 | RPL10A | hypothetical protein PANDA_002233, partial [*Ailuropoda melanoleuca*] | 0.0005 | -12.1755 |
| 22933 | SIRT2 | NAD-dependent protein deacetylase sirtuin-2 isoform X3 [*Rattus norvegicus*] | 0.0223 | -12.0249 |
| 8851 | CDK5R1 | cyclin-dependent kinase 5 activator 1 | 0.0224 | -11.9929 |
| 386675 | KRTAP10-7 | *Cricetulus griseus* protein kinase C, alpha (Prkca), transcript variant X1, mRNA | 0.0048 | -11.4560 |
| 84893 | FBXO18 | F-box DNA helicase 1 isoform X1 [*Peromyscus maniculatus bairdii*] | 0.0217 | -11.0912 |
| **80333** | **KCNIP4** | **Kv channel-interacting protein 4 isoform 4 [*Homo sapiens*]** | **0.0229** | **-11.0836** |
| 83733 | SLC25A18 | mitochondrial glutamate carrier 2 | 0.0189 | -11.0658 |
| 22826 | DNAJC8 | dnaJ homolog subfamily C member 8 | 2.84E-05 | -11.0570 |
| 1741 | DLG3 | disks large homolog 3 isoform 2 | 0.0189 | -11.0524 |
| 79411 | GLB1L | beta-galactosidase-1-like protein isoform X1 [*Peromyscus maniculatus bairdii*] | 0.0219 | -10.8621 |
| 51629 | SLC25A39 | solute carrier family 25 member 39 [*Peromyscus maniculatus bairdii*] | 0.0211 | -10.8334 |
| 55605 | KIF21A | kinesin-like protein KIF21A [Peromyscus maniculatus bairdii] | 0.0211 | -10.8222 |
| 6196 | RPS6KA2 | ribosomal protein S6 kinase alpha-2 isoform X2 [*Peromyscus maniculatus bairdii*] | 9.40E-9 | -10.7820 |
| 322 | APBB1 | amyloid beta A4 precursor protein-binding family B member 1 isoform X3 [*Peromyscus maniculatus bairdii*] | 0.0015 | -10.7406 |
| 22919 | MAPRE1 | Mapre1 protein, partial *[Mus musculus]* | 0.0235 | -10.5714 |
| 56853 | CELF4 | *Peromyscus maniculatus bairdii* CUGBP, Elav-like family member 4 (Celf4), transcript variant X2, mRNA | 0.0234 | -10.5435 |
| 4043 | LRPAP1 | alpha-2-macroglobulin receptor-associated protein [*Peromyscus maniculatus bairdii*] | 0.0254 | -10.4091 |
| 138046 | RALYL | RNA-binding Raly-like protein isoform X2 [*Rattus norvegicus*] | 0.0373 | -10.3605 |
| 322 | APBB1 | amyloid beta A4 precursor protein-binding family B member 1 isoform X1 [*Jaculus jaculus*] | 0.0263 | -10.3515 |
