# Supplementary material for: Hypothalamic transcriptomic alterations in male and female California mice (Peromyscus californicus) developmentally exposed to bisphenol A or ethinyl estradiol
Source: Physiol Rep. 2017 Feb 14;5(3):e13133. doi: 10.14814/phy2.13133 (PMC5309579; doi:10.14814/phy2.13133)
Supplement: Supplementary file 2 — Table S2. Top 20 annotated genes upregulated in control males compared to control females. Shaded row is included in the BPA group (Table 4). [file PHY2-5-e13133-s002.docx]

| **Supplementary Table 2**. Top 20 annotated genes up regulated in control males compared to control females. Shaded row is also included in the BPA group (Table 4). | | | | |
| --- | --- | --- | --- | --- |
| **Entrez ID** | **Gene Symbol** | **Gene Name** | **FDR** | **Log2 Fold Change** |
| 4301 | MLLT4 | afadin isoform X14 [*Mus musculus*] | 0.0052 | 12.2078 |
| 55573 | CDV3 | protein CDV3 homolog isoform X2 [*Marmota marmota marmota*] | 7.11E-8 | 11.9696 |
| 826 | CAPNS1 | calpain small subunit 1 [*Peromyscus maniculatus bairdii*] | 0.0005 | 11.8586 |
| 5609 | MAP2K7 | dual specificity mitogen-activated protein kinase kinase 7 [*Rattus norvegicus*] | 0.0005 | 11.6814 |
| 54841 | BIVM | *Peromyscus maniculatus bairdii* uncharacterized *LOC102921188 (LOC102921188), ncRNA* | 0.0182 | 11.2099 |
| 10659 | CELF2 | CUGBP Elav-like family member 2 isoform X6 [*Cricetulus griseus*] | 4.55E-7 | 11.1992 |
| NULL | N/A | *Cricetulus griseus* cytidine monophosphate (UMP-CMP) kinase 1, cytosolic (Cmpk1), mRNA | 0.0128 | 10.9589 |
| 26035 | GLCE | D-glucuronyl C5-epimerase [*Peromyscus maniculatus bairdii*] | 8.27E-10 | 10.8875 |
| 2548 | GAA | lysosomal alpha-glucosidase isoform X1 *[Peromyscus maniculatus bairdii]* | 0.0229 | 10.8050 |
| 25842 | ASF1A | LOW QUALITY PROTEIN: histone chaperone ASF1A [*Pan troglodytes*] | 0.0011 | 10.7629 |
| 6728 | SRP19 | *Peromyscus maniculatus bairdii* signal recognition particle 19kDa (Srp19), mRNA | 1.13E-6 | 10.7033 |
| 80333 | KCNIP4 | Kv channel-interacting protein 4 isoform 2 [Homo sapiens] | 0.0019 | 10.5810 |
| 63971 | KIF13A | kinesin-like protein KIF13A isoform X4 [*Peromyscus maniculatus bairdii*] | 0.0436 | 10.4608 |
| 9755 | TBKBP1 | TANK-binding kinase 1-binding protein 1 [*Peromyscus maniculatus bairdii*] | 0.0139 | 10.4514 |
| 7453 | WARS | tryptophan--tRNA ligase, cytoplasmic [*Peromyscus maniculatus bairdii*] | 0.0002 | 10.4485 |
| 64400 | AKTIP | AKT-interacting protein [*Peromyscus maniculatus bairdii*] | 0.0139 | 10.3611 |
| 4311 | MME | neprilysin [*Peromyscus maniculatus bairdii*] | 0.0005 | 10.2364 |
| 100506658 | OCLN | occludin [*Peromyscus maniculatus bairdii*] | 0.0162 | 10.0295 |
| 255520 | ELMOD2 | ELMO domain-containing protein 2 [*Peromyscus maniculatus bairdii*] | 1.96E-5 | 9.8856 |
| 346157 | ZNF391 | zinc finger protein 282-like [*Peromyscus maniculatus bairdii]* | 0.0020 | 9.8347 |
