# Supplementary material for: Hypothalamic transcriptomic alterations in male and female California mice (Peromyscus californicus) developmentally exposed to bisphenol A or ethinyl estradiol
Source: Physiol Rep. 2017 Feb 14;5(3):e13133. doi: 10.14814/phy2.13133 (PMC5309579; doi:10.14814/phy2.13133)
Supplement: Supplementary file 3 — Table S3. Top 20 annotated genes downregulated in BPA males compared to BPA females. Shaded row is included in the Control group (Table S1). [file PHY2-5-e13133-s003.docx]

| **Supplementary Table 3**. Top 20 annotated genes down regulated in BPA males compared to BPA females. Shaded row is also included in the Control group (Supplementary Table 1). | | | | |
| --- | --- | --- | --- | --- |
| **Entrez ID** | **Gene Symbol** | **Gene Name** | **FDR** | **Log2 Fold Change** |
| 23008 | KLHDC10 | kelch domain-containing protein 10 | 0.0460 | -13.0684 |
| 157378 | TMEM65 | transmembrane protein 65 | 0.0050 | -12.6194 |
| 55831 | EMC3 | ER membrane protein complex subunit 3 | 0.0003 | -11.9025 |
| 10210 | TOPORS | *Mustela putorius* furo UDP-Gal:betaGlcNAc beta 1,4- galactosyltransferase, polypeptide 5 (B4GALT5), partial mRNA | 0.01367 | -11.8296 |
| 57680 | CHD8 | chromodomain-helicase-DNA-binding protein 8 isoform X5 | 0.0130 | -11.6454 |
| 9107 | MTMR6 | myotubularin-related protein 6 isoform X1 | 3.12E-06 | -11.2861 |
| 24139 | EML2 | LOW QUALITY PROTEIN: echinoderm microtubule-associated protein-like 2 [*Peromyscus maniculatus bairdii*] | 0.0009 | -10.8534 |
| 137695 | TMEM68 | transmembrane protein 68 | 2.17E-06 | -10.8070 |
| 51631 | LUC7L2 | LUC7-like 2 | 0.0104 | -10.4275 |
| 56853 | CELF4 | *Peromyscus maniculatus bairdii* CUGBP, Elav-like family member 4 (Celf4), transcript variant X5, mRNA | 0.0008 | -10.4275 |
| 493856 | CISD2 | *Peromyscus maniculatus bairdii* CDGSH iron sulfur domain 2 (Cisd2), mRNA | 0.0021 | -10.3336 |
| 55705 | IPO9 | mKIAA1192 protein [*Mus musculus*] | 0.0022 | NULL |
| 56980 | PRDM10 | PR domain zinc finger protein 10 isoform X4 | 9.83E-15 | -10.1859 |
| 129138 | ANKRD54 | ankyrin repeat domain-containing protein 54 | 0.0125 | -10.1032 |
| 25847 | ANAPC13 | *Peromyscus maniculatus bairdii* anaphase promoting complex subunit 13 (Anapc13), transcript variant X3, mRNA | 0.0368 | -9.9954 |
| 22839 | DLGAP4 | disks large-associated protein 4 isoform X1 [*Peromyscus maniculatus bairdii*] | 0.0024 | -9.9539 |
| 5274 | SERPINI1 | neuroserpin precursor | 0.0064 | -9.9521 |
| 8899 | PRPF4B | Serine/threonine- protein kinase | 0.0330 | -9.9176 |
| 196463 | PLBD2 | putative phospholipase B-like 2 [*Peromyscus maniculatus bairdii*] | 0.0038 | -9.8947 |
| 5136 | PDE1A | calcium/calmodulin-dependent 3',5'-cyclic nucleotide phosphodiesterase 1A isoform X1 | 0.0437 | -9.7873 |
