# Supplementary material for: Hypothalamic transcriptomic alterations in male and female California mice (Peromyscus californicus) developmentally exposed to bisphenol A or ethinyl estradiol
Source: Physiol Rep. 2017 Feb 14;5(3):e13133. doi: 10.14814/phy2.13133 (PMC5309579; doi:10.14814/phy2.13133)
Supplement: Supplementary file 4 — Table S4. Top 20 annotated genes upregulated in BPA males compared to BPA females. Shaded row is listed in controls (Table S2). [file PHY2-5-e13133-s004.docx]

| **Supplementary Table 4**. Top 20 annotated genes up regulated in BPA males compared to BPA females. Shaded row is also listed in Controls (Supplementary Table 2). | | | | |
| --- | --- | --- | --- | --- |
| **Entrez ID** | **Gene Symbol** | **Gene Name** | **FDR** | **Log2 Fold Change** |
| 65125 | WNK1 | serine/threonine-protein kinase WNK1 isoform X3 [*Cricetulus griseus*] | 0.0158 | 12.7111 |
| 170506 | DHX36 | ATP-dependent RNA helicase DHX36 | 0.0089 | 12.6299 |
| 8242 | KDM5C | lysine-specific demethylase 5D isoform X1 | 6.82E-32 | 12.4275 |
| 8284 | KDM5D | lysine-specific demethylase 5D isoform X2 [*Mus musculus*] | 2.19E-27 | 12.0444 |
| 5909 | RAP1GAP | rap1 GTPase-activating protein 1 isoform X13 [*Peromyscus maniculatus bairdii*] | 5.48E-07 | 11.9354 |
| 23683 | PRKD3 | serine/threonine-protein kinase D3 isoform X2 [*Peromyscus maniculatus bairdii*] | 1.80E-06 | 10.6999 |
| 3631 | INPP4A | type I inositol 3,4-bisphosphate 4-phosphatase isoform X3 [*Peromyscus maniculatus bairdii*] | 0.0303 | 10.5505 |
| 2222 | FDFT1 | squalene synthase [*Mesocricetus auratus*] | 0.0413 | 10.5180 |
| 9755 | TBKBP1 | TANK-binding kinase 1-binding protein 1 [*Peromyscus maniculatus bairdii*] | 0.0317 | 10.4691 |
| 572 | BAD | bcl2-associated agonist of cell death isoform X1 [*Peromyscus maniculatus bairdii*] | 0.0331 | 10.3331 |
| 23031 | MAST3 | microtubule-associated serine/threonine-protein kinase 3 isoform X4 [*Peromyscus maniculatus bairdii*] | 0.0377 | 10.2905 |
| 3752 | KCND3 | potassium voltage-gated channel subfamily D member 3 isoform 2 precursor [*Homo sapiens*] | 0.0409 | 10.2805 |
| 2058 | EPRS | bifunctional glutamate/proline--tRNA ligase isoform X2 [*Mus musculus*] | 0.0375 | 10.2734 |
| 7145 | TNS1 | tensin-1 isoform X4 [*Peromyscus maniculatus bairdii*] | 0.0033 | 10.2017 |
| 617 | BCS1L | mitochondrial chaperone BCS1 [*Rattus norvegicus*] | 0.0350 | 10.1838 |
| 51447 | IP6K2 | uncharacterized protein LOC460352 isoform X8 [*Pan troglodytes*] | 0.0368 | 10.1410 |
| 8242 | KDM5C | lysine-specific demethylase 5C isoform X3 [*Peromyscus maniculatus bairdii*] | 5.62E-29 | 10.1240 |
| 51231 | VRK3 | inactive serine/threonine-protein kinase VRK3 [*Peromyscus maniculatus bairdii*] | 0.0415 | 10.0199 |
| 10507 | SEMA4D | semaphorin-4D isoform X4 [*Peromyscus maniculatus bairdii*] | 0.0008 | 9.9718 |
| 2551 | GABPA | GA-binding protein alpha chain isoform X2 [*Peromyscus maniculatus bairdii*] | 0.0409 | 9.9535 |
