# Supplementary material for: Hypothalamic transcriptomic alterations in male and female California mice (Peromyscus californicus) developmentally exposed to bisphenol A or ethinyl estradiol
Source: Physiol Rep. 2017 Feb 14;5(3):e13133. doi: 10.14814/phy2.13133 (PMC5309579; doi:10.14814/phy2.13133)
Supplement: Supplementary file 5 — Table S5. Top 20 annotated genes downregulated in EE males compared to EE females. Bold row is included in the control group (Table S1). [file PHY2-5-e13133-s005.docx]

| **Supplementary Table 5**. Top 20 annotated genes down regulated in EE males compared to EE females. Bolded row is also included in the Control group (Supplementary Table 1). | | | | |
| --- | --- | --- | --- | --- |
| **Entrez ID** | **Gene Symbol** | **Gene Name** | **FDR** | **Log2 Fold Change** |
| 8851 | CDK5R1 | cyclin-dependent kinase 5 activator 1 [*Mus musculu*s] | 0.0004 | -13.2392 |
| 4232 | MEST | mesoderm-specific transcript homolog protein isoform X2 [*Peromyscus maniculatus bairdii*] | 0.0003 | -12.9036 |
| 51747 | LUC7L3 | luc7-like protein 3 isoform X1 [*Microtus ochrogaster*] | 0.0347 | -12.6646 |
| 11237 | RNF24 | RING finger protein 24 [*Cricetulus griseus*] | 0.0058 | -12.4595 |
| **80333** | **KCNIP4** | **Kv channel-interacting protein 4 isoform 4 [*Homo sapiens*]** | **0.0489** | **-12.0452** |
| 22866 | CNKSR2 | connector enhancer of kinase suppressor of ras 2 isoform X4 [*Peromyscus maniculatus bairdii*] | 0.0036 | -11.3153 |
| 152926 | PPM1K | protein phosphatase 1K, mitochondrial [*Peromyscus maniculatus bairdii*] | 0.0223 | -11.2257 |
| 8266 | UBL4A | *Peromyscus maniculatus bairdii* ubiquitin-like 4A (Ubl4a), mRNA | 0.0499 | -11.1700 |
| 10147 | SUGP2 | SURP and G-patch domain-containing protein 2 isoform X3 [*Peromyscus maniculatus bairdii*] | 0.0066 | -10.7624 |
| 10743 | RAI1 | retinoic acid-induced protein 1 [*Peromyscus maniculatus bairdii*] | 0.0097 | -10.4353 |
| 1040 | CDS1 | phosphatidate cytidylyltransferase 1 [*Callorhinchus milii*] | 0.0158 | -9.9543 |
| 6654 | SOS1 | son of sevenless homolog 1 [*Microtus ochrogaster*] | 0.0436 | -9.9394 |
| 166336 | PRICKLE2 | prickle-like protein 2 isoform X1 [*Peromyscus maniculatus bairdii*] | 0.0406 | -9.9080 |
| 5893 | RAD52 | DNA repair protein RAD52-like [*Cricetulus griseus*] | 0.0002 | -9.7827 |
| 4987 | OPRL1 | nociceptin receptor isoform 1x | 4.39E-05 | -9.7173 |
| 10802 | SEC24A | protein transport protein Sec24A [*Peromyscus maniculatus bairdii*] | 0.0259 | -9.6490 |
| 126119 | JOSD2 | josephin-2 isoform X1 [*Cricetulus griseus*] | 0.0198 | -9.6083 |
| 55051 | NRDE2 | protein NRDE2 homolog isoform X4 [*Peromyscus maniculatus bairdii*] | 0.0227 | -9.5625 |
| 26284 | ERAL1 | GTPase Era, mitochondrial [*Peromyscus maniculatus bairdii*] | 0.0003 | -9.4294 |
| 57504 | MTA3 | metastasis-associated protein MTA3 isoform X2 [*Cricetulus griseus*] | 0.0259 | -9.3724 |
