# Supplementary material for: Hypothalamic transcriptomic alterations in male and female California mice (Peromyscus californicus) developmentally exposed to bisphenol A or ethinyl estradiol
Source: Physiol Rep. 2017 Feb 14;5(3):e13133. doi: 10.14814/phy2.13133 (PMC5309579; doi:10.14814/phy2.13133)
Supplement: Supplementary file 6 — Table S6. Top 20 annotated genes upregulated in EE males compared to EE females. [file PHY2-5-e13133-s006.docx]

| **Supplementary Table 6**. Top 20 annotated genes up regulated in EE males compared to EE females. | | | | |
| --- | --- | --- | --- | --- |
| **Entrez ID** | **Gene Symbol** | **Gene Name** | **FDR** | **Log2 Fold Change** |
| 9524 | TECR | very-long-chain enoyl-CoA reductase isoform 2 | 0.0053 | 14.0920 |
| 157378 | TMEM65 | transmembrane protein 65 [*Microtus ochrogaster*] | 0.0085 | 13.3165 |
| 6507 | SLC1A3 | excitatory amino acid transporter 1 isoform 4 [*Rattus norvegicus*] | 0.0076 | 13.2750 |
| 51629 | SLC25A39 | solute carrier family 25 member 39 [*Peromyscus maniculatus bairdii*] | 5.41E-05 | 12.5884 |
| 10390 | CEPT1 | choline/ethanolaminephosphotransferase 1 isoform X2 *[Peromyscus maniculatus bairdii]* | 0.0004 | 12.5289 |
| 57701 | NCKAP5L | *Microtus ochrogaster* chromosome LG3 open reading frame, human C7orf41 (CLG3H7orf41), mRNA | 0.0029 | 12.4113 |
| 2036 | EPB41L1 | band 4.1-like protein 1 isoform X12 [*Peromyscus maniculatus bairdii*] | 0.0137 | 12.3838 |
| 84918 | LRP11 | low-density lipoprotein receptor-related protein 11 [*Peromyscus maniculatus bairdii*] | 0.0164 | 12.3226 |
| 5007 | OSBP | oxysterol-binding protein 1 isoform X1 *[Rattus norvegicus]* | 0.0178 | 12.2126 |
| 16 | AARS | alanine--tRNA ligase, cytoplasmic [*Peromyscus maniculatus bairdii*] | 0.0160 | 12.1891 |
| 4826 | NNAT | *Peromyscus maniculatus bairdii* neuronatin (Nnat), transcript variant X2, mRNA | **0.0166** | **12.1456** |
| 55074 | OXR1 | oxidation resistance protein 1 isoform X3 [*Peromyscus maniculatus bairdii*] | 0.0005 | 12.1098 |
| 2222 | FDFT1 | squalene synthase [*Mesocricetus auratus*] | 0.0213 | 11.7796 |
| 9026 | HIP1R | huntingtin-interacting protein 1-related protein isoform X1 [*Mesocricetus auratus*] | 0.0242 | 11.7338 |
| 80306 | MED28 | *Peromyscus maniculatus bairdii* mediator complex subunit 28 (Med28), mRNA | 0.0039 | 11.5995 |
| 780 | DDR1 | epithelial discoidin domain-containing receptor 1 isoform X2 [*Peromyscus maniculatus bairdii*] | 0.0247 | 11.5310 |
| 29097 | CNIH4 | protein cornichon homolog 4 [*Mus musculus*] | 0.0005 | 11.4424 |
| 9659 | PDE4DIP | myomegalin-like isoform X13 [*Peromyscus maniculatus bairdii*] | 0.0284 | 11.3620 |
| 132204 | SYNPR | synaptoporin precursor [*Rattus norvegicus*] | 0.0301 | 11.2826 |
| 221037 | JMJD1C | probable JmjC domain-containing histone demethylation protein 2C isoform X1 [*Peromyscus maniculatus bairdii*] | 0.0308 | 11.2617 |
