# Supplementary material for: Hypothalamic transcriptomic alterations in male and female California mice (Peromyscus californicus) developmentally exposed to bisphenol A or ethinyl estradiol
Source: Physiol Rep. 2017 Feb 14;5(3):e13133. doi: 10.14814/phy2.13133 (PMC5309579; doi:10.14814/phy2.13133)
Supplement: Supplementary file 10 [file PHY2-5-e13133-s010.docx]

Data S1: *Genes Increased or Decreased in Both BPA- and EE-Exposed Groups*.

Data S2: *Differentially Expressed Genes Based on Treatment and Sex*.

Data S3: *Differentially Expressed Genes Based on Treatment*.
